# Synthesis and full-spectrum-responsive photocatalytic activity from UV/Vis to near-infrared region of S-O decorated YMnO_3_ nanoparticles for photocatalytic degradation of ibuprofen

**DOI:** 10.3389/fchem.2024.1424548

**Published:** 2024-06-07

**Authors:** Zixia Feng

**Affiliations:** Faculty of Science, Hong Kong University of Science and Technology, Hong Kong, Hong Kong SAR, China

**Keywords:** YMnO3, ibuprofen, photocatalytic activity, hole, superoxide free radical

## Abstract

The oxalic acid complexation method and sulfuric acid heat treatment method were used to synthesize the YMnO_3_ (YMO) and YMO-SO_4_^2-^ (YMO-SO) photocatalysts. The YMO-SO photocatalyst maintained the crystal structure of YMO, but the particle size increased slightly and the optical band gap decreased significantly. The YMO-SO photocatalyst demonstrates a wide range of light absorption capabilities, covering ultraviolet, visible and near-infrared light. The photocatalytic activity of YMO-SO was investigated with ibuprofen as the target pollutant. The YMO-SO photocatalyst exhibits high ultraviolet (UV), visible and near-infrared photocatalytic activity. Experiments with different environmental parameters confirmed that the best catalyst content was 1 g/L, the best drug concentration was 75 mg/L and the best pH value was 7. The capture experiment, free radical detection experiment and photocatalytic mechanism analysis confirmed that the main active species of YMO-SO photocatalyst were hole and superoxide free radical.

## 1 Introduction

The water system is contaminated with ibuprofen, which is currently the third most consumed drug in the world, due to human drug use or improper disposal of expired drugs (Osman et al., 2024). For such pollutants, it is necessary to degrade them from the source in order to effectively eliminate their pollution to the environment (Othman et al., 2024). To effectively degrade ibuprofen, researchers have used a number of methods to try to remove this contaminant, including adsorption, electrocatalysis, photocatalysis, thermal catalysis, and biodegradation (Navrozidou et al., 2019; Falletta et al., 2023; Abdo et al., 2024; Jin et al., 2024; Li et al., 2024). Among these methods, photocatalysis is a green technology that degrades ibuprofen only with the help of light energy (Sruthi et al., 2021; Rebollo et al., 2024). Although it is said that light energy is required, the degradation of ibuprofen cannot be completed without a photocatalyst (Shakir, 2024). Therefore, the development of photocatalysts for the efficient use of sunlight to degrade ibuprofen has become the key.

Yttrium manganate (YMnO_3_, YMO) is a common photocatalyst that has been used to degrade dyes due to its high specific surface area, high charge transfer and separation efficiency, high thermal and chemical stability (Wang et al., 2011; Kumar et al., 2019; López-Alvarez et al., 2023). Although it is effective at degrading dyes, a single component of YMO has not been used to degrade contaminants such as pharmaceuticals. To further improve the photocatalytic activity of YMO, researchers mainly adopted two effective ways to improve its photocatalytic activity. (1) The optical band gap value of YMO was improved by ion doping, and its photocatalytic activity was also increased (Zhang et al., 2011; Munisha et al., 2023). (2) The photocatalytic activity of YMO was enhanced by combining with other semiconductors with excellent photocatalytic performance (Cao et al., 2018; Wu et al., 2019; Wang and Song, 2020; Wang and Tian, 2020; You et al., 2020; Wang et al., 2022; Zhang et al., 2022; Li et al., 2023; Wang et al., 2023; Yulizar et al., 2023; Dang et al., 2024). However, no one has yet synthesized SO_4_^2-^ surface decorated YMO (YMO-SO) photocatalyst to enhance the photocatalytic activity of YMO by a simple heat-treating YMO with sulfuric acid. Therefore, it is of great significance to study the physicochemical properties of YMO by a simple heat-treating with sulfuric acid. Notably, YMO exhibits high optical absorption coefficients across the full spectral range from UV-visible to near-infrared light (Wang et al., 2011; Kumar et al., 2019; López-Alvarez et al., 2023). At present, YMO as a photocatalyst has been used to photodegrade dyes or antibiotics under ultraviolet and visible light irradiation, but there are no reports of near-infrared photocatalysis. Simultaneously, YMO has been used to degrade tetracycline hydrochloride and oxytetracycline hydrochloride, but it has not been reported that YMO is used to degrade ibuprofen. Therefore, the synthesis of YMO-SO photocatalyst by a special method and its use in the degradation of ibuprofen in the full spectral range will provide guidance for other similar studies.

In this paper, we propose to synthesize YMO photocatalyst by oxalic acid complexation method, and then heat treat YMO with sulfuric acid to obtain YMO-SO photocatalyst. A variety of characterization methods were used to determine whether the crystal structure, surface morphology and color properties of YMO were changed after special treatment. Meanwhile, the effects of different illumination conditions, including ultraviolet light, visible light and near-infrared light, on the photocatalytic activity of YMO and YMO-SO photocatalysts for the degradation of ibuprofen were investigated. The effects of different environmental parameters, including catalyst content, drug concentration and pH value, on the photocatalytic activity of YMO and YMO-SO photocatalysts have also been deeply explored. The contribution of active species to the degradation of ibuprofen by YMO and YMO-SO photocatalysts was explored through capture experiments and free radical validation experiments.

## 2 Experiment

### 2.1 Preparation of YMO and YMO-SO photocatalyst

**FIGURE 1:**
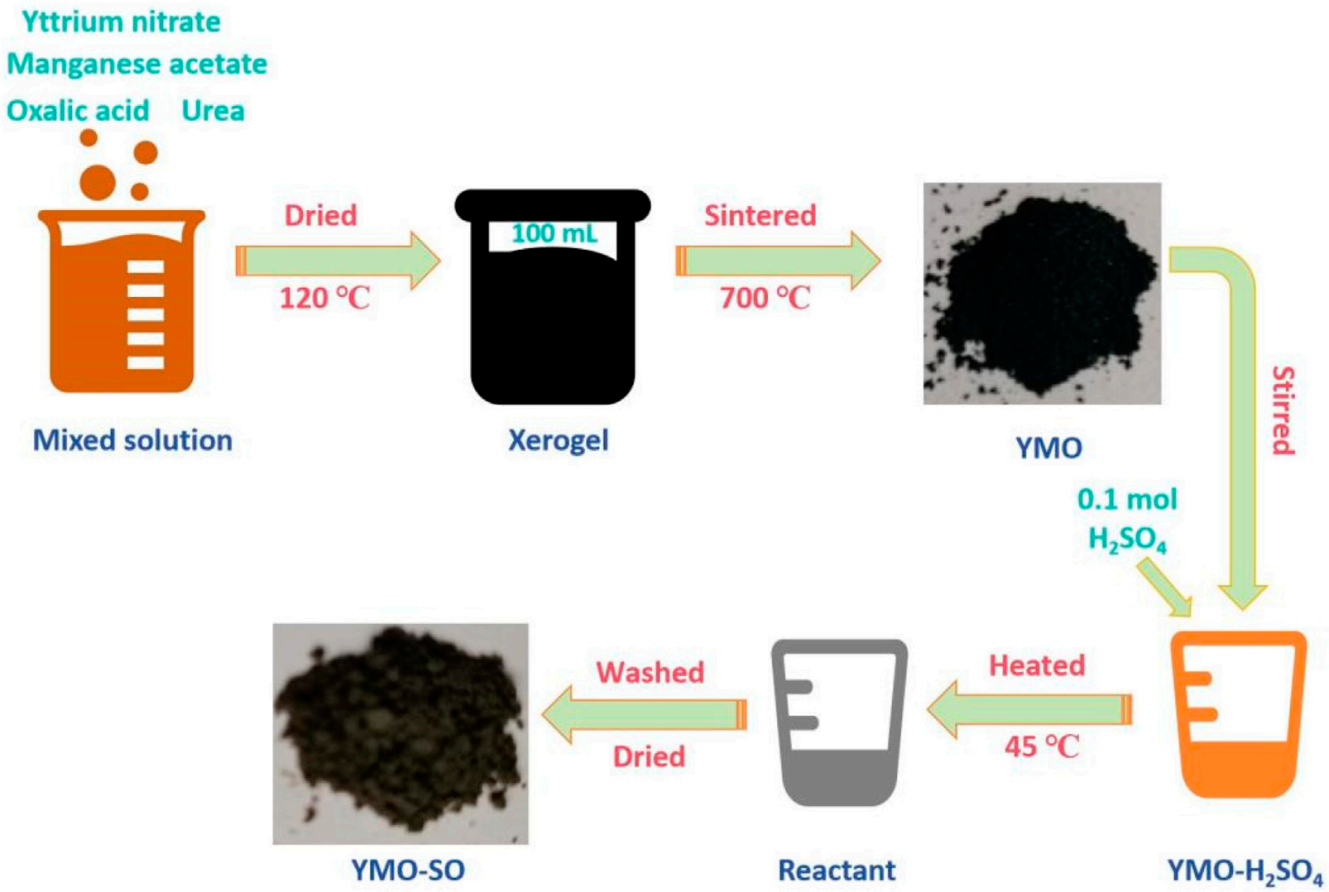
Preparation flow chart of the YMO and YMO-SO.

Appropriate amount of yttrium nitrate and manganese acetate were weighed and dissolved in 40 mL of deionized water according to the molar ratio of Y: Mn = 1 : 1. After the reagent is completely dissolved, 5 g of weighed oxalic acid is added as a complexing agent to remove the metal ions in the reaction solution. After the complete reaction of oxalic acid, 15 g urea was added to participate in the reaction. After stirring thoroughly for 3 h, the mixed solution is obtained. Then, stir on the heating side of the magnetic stirrer for 2 h at a heating temperature of 90°C. After obtaining the viscous yellowish brown colloid, it was placed in a drying oven and dried at 120°C for 48 h to obtain xerogel. The dry gel was cooled and ground, and then placed in the sintering furnace at 700°C for 5 h to obtain YMnO_3_ (YMO) powder. Part of the obtained YMO powder was dispersed in alcohol solution for ultrasound for 20 min. Let stand for 10 h, then pour out the supernatant and add 10 mL of deionized water. Then, 0.1 mol/L concentrated sulfuric acid was added to react for 24 h under the action of magnetic stirring. Meanwhile, heat to 45°C for heat treatment. After the reaction was completed, the powder was rinsed with deionized water for 3 times and dried in a drying oven at 120°C for 1 h to obtain YMO-SO_4_^2-^ (YMO-SO) powder. Figure 1 shows the preparation flow chart of the YMO and YMO-SO.

### 2.2 Materials characterization

The phase structure of the YMO and YMO-SO were measured by a D8 ADVANCE X-ray powder diffractometer (XRD) with the working voltage of 30 kV and the working current of 40 mA. The Fourier infrared (FTIR) spectra of the YMO and YMO-SO were measured by a FTIR-650 Fourier infrared spectrometer with the wave number range of 400–1,000 cm^-1^. The microstructure characteristics of the YMO and YMO-SO were characterized by a Themo Scientific Axia Chemi field emission scanning electron microscopy (SEM) and JEM 2100F transmission electron microscopy (TEM). The UV-visible diffuse reflectance spectrum of the YMO and YMO-SO were characterized by a UV1901 UV-visible spectrophotometer with the BaSO_4_ as reference.

### 2.3 Photocatalytic experiments

To perform the photocatalytic experiment of the YMO and YMO-SO, ibuprofen was selected as the target degradation pollutant. The initial ibuprofen concentration, catalyst content and reaction solution pH values were 25–125 mg/L, 0.5–1.5 g/L, 3–11, respectively. The photocatalytic equipment uses the products of Porfilet Technology Co., Ltd. and the irradiation light source is 300 W xenon lamp by adding filters to obtain ultraviolet light, visible light and near-infrared light. The prepared reaction solution (100 mL) is placed on the mixer and continuously stirred magnetically. Before photocatalysis, the adsorption experiment was performed for half an hour to eliminate the effect of adsorption on photocatalysis. After half an hour of adsorption experiment, xenon lamp light source was turned on and photocatalytic experiment was performed. Simultaneously, the effects of ultraviolet light, visible light and near-infrared light on the photocatalytic activity of the photocatalyst were studied by adding filters to the photocatalytic equipment. Every 10 minutes, take the reaction solution for 5 mL until 1 h. The reaction solution was centrifuged, and the supernatant was taken for concentration measurement experiment, and the concentration data were recorded. The degradation percentage (D%) of ibuprofen is defined as (*C*_0_ − *C*_t_)/*C*_0_, where *C*_0_ and *C*_t_ are the concentration values of ibuprofen before and after irradiation, respectively. To explore the role of free radicals in the photocatalysis process, capture experiments were performed. The disodium ethylenediamine tetraacetic acid (EDTA-2Na), 2-propanol (IPA) and 1, 4-benzoquinone (BQ) were used as scavengers to detect the holes (h_VB_^+^), hydroxyl radicals (•OH) and superoxide radicals (•O_2_^−^), respectively. During the capture experiment, except for adding 1 mmol of trapping agent to the reaction solution, other processes were consistent with the photocatalytic experiment. The h_VB_^+^, •OH and •O_2_^−^ can be also confirmed by a Bruker A300 spectrometer for the electron spin resonance (ESR)/electron paramagnetic resonance (EPR) experiments.

## 3 Results and discussion

### 3.1 Phase structure and purity analysis

**FIGURE 2:**
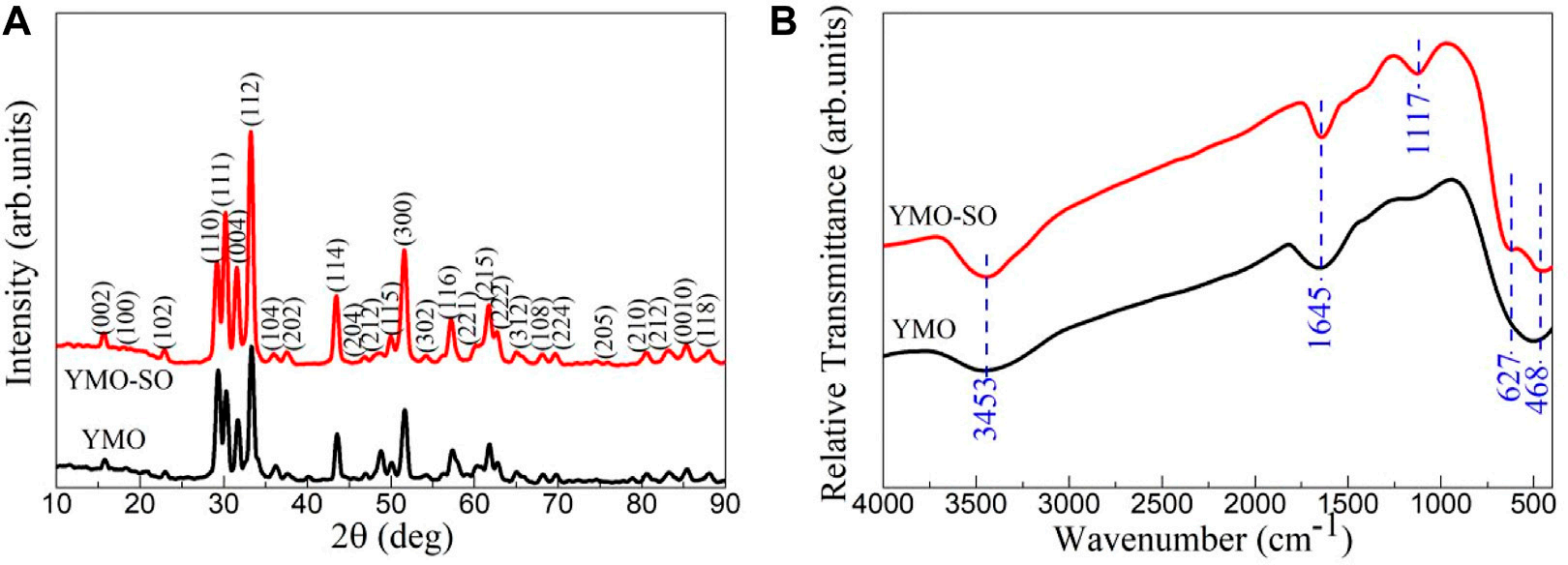
**(A)** XRD pattern and **(B)** FTIR spectra of the YMO and YMO-SO.

The phase structure and purity of photocatalyst have great influence on its photocatalytic activity. To explore the effect of heat treatment of YMO with sulfuric acid on its phase structure and purity, XRD patterns and FTIR spectra are shown in Figure 2. Figure 2A displays the XRD pattern of the YMO and YMO-SO. The diffraction peaks of the YMO and YMO-SO can be indexed in terms of the hexagonal phase of YMO with the standard JCPDS file no. 25–1,079. The crystallite sizes of the YMO and YMO-SO were estimated by the Scherrer Eq. 1:

$$D=\frac{k\lambda }{\beta \cos\theta }$$ (1)

Where, k is the shape factor and k = 0.9, λ is the wavelength of the incident X-rays and λ = 0.15406 nm, β is the full width of diffraction peak and θ is the Bragg angle of the diffraction peak. The crystallite sizes of the YMO and YMO-SO are 35 and 43 nm, respectively. After heat treatment with sulfuric acid, the main reason for the increase in crystallite size may be that the cleaning after heat treatment makes the finer YMO particles flow out with the cleaning solution. To explore the functional groups contained in YMO and YMO-SO, detailed characterization by FTIR spectroscopy is required.

Figure 2B displays the FTIR spectra of the YMO and YMO-SO. For all samples, four obvious characteristic peaks at 3,453, 1,645, 627, and 468 cm^−1^ can be observed. The peaks at 3,453 and 1,645 cm^−1^ can be assigned to the stretching vibration of O-H and the bending vibration of H-O-H of absorbed water, respectively (Wu et al., 2019; Zhang et al., 2022; Dang et al., 2024; Zhu et al., 2024). The presence of adsorbed water is mainly due to the absorption of water by the sample during storage and the use of potassium bromide during testing. The peaks at 627 and 468 cm^-1^ can be ascribed to the Y-O stretching and Mn-O-Mn bending vibrations, respectively (Zhang and Wang, 2017; Shukla et al., 2023). In addition, a new characteristic peak at 1,117 cm^−1^ appeared in the YMO-SO sample. The peak can be attributed to the S-O bond (Ramesh et al., 2008; Tong et al., 2010). This result further confirms that the S-O bond has been decorated on the surface of YMO after heat treatment with sulfuric acid.

### 3.2 Microstructural analysis

**FIGURE 3:**
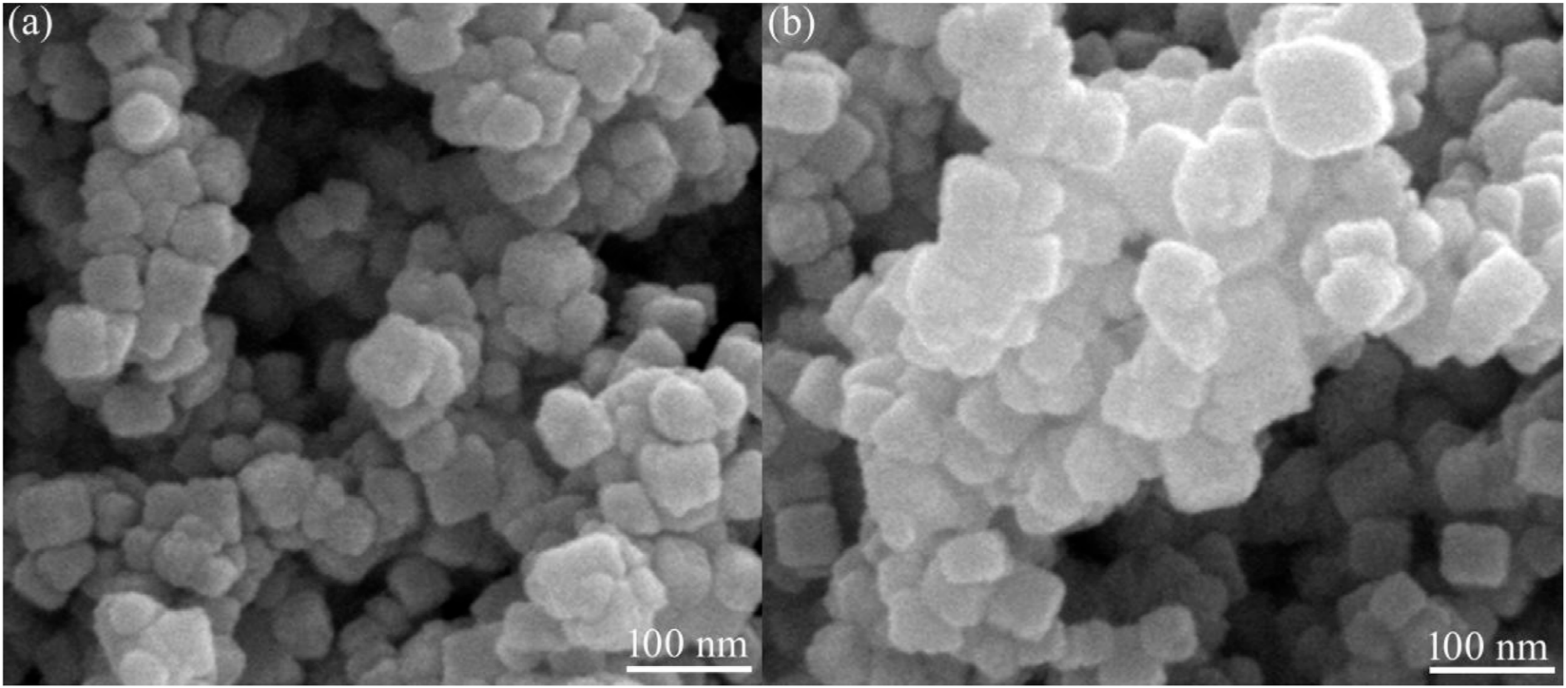
SEM images of the **(A)** YMO and **(B)** YMO-SO.

The crystallite sizes of YMO and YMO-SO were calculated by Scherrer’s formula, and YMO-SO showed a larger crystallite size than YMO. To further confirm this phenomenon, SEM observations were performed. Figure 3 displays the SEM images of the YMO and YMO-SO. The YMO particles prepared by a simple oxalic acid complexation method were fine, uniform and approximately spherical as shown in Figure 3A. The average particle size of the particles is about 30 nm, and there is agglomeration among the particles. Figure 3B displays the SEM image of the YMO-SO. The phenomenon of particle growth is very obvious, and the surface of the particle becomes more rough. Another reason for the larger particle size of YMO-SO may be that the S-O functional group is decorated on the surface of YMO, which makes the particle size of YMO-SO increase. The average particle size of the YMO-SO is about 40 nm. The results observed by SEM are similar to those calculated by XRD.

**FIGURE 4:**
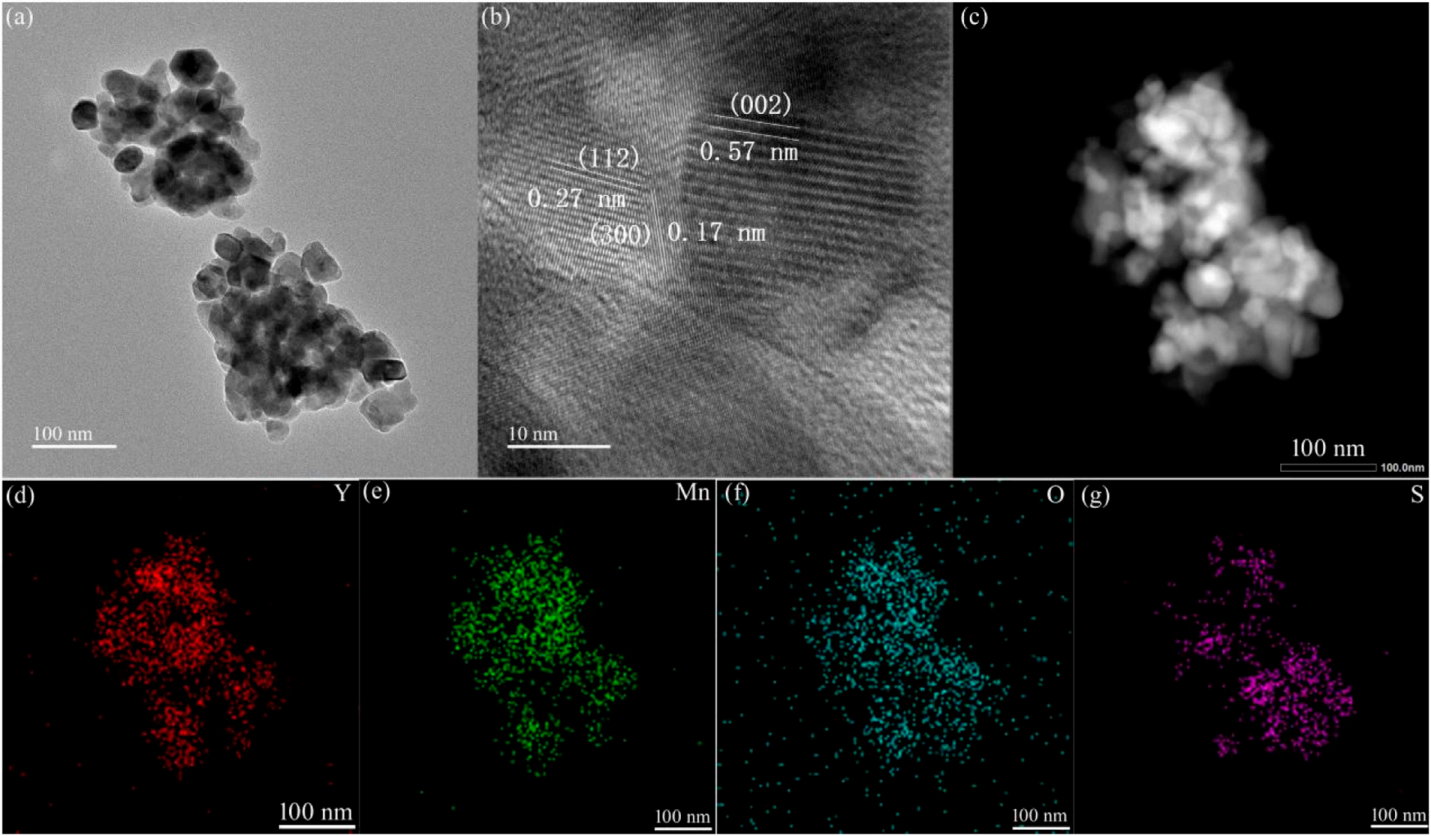
**(A)** TEM, **(B)** HRTEM, **(C)** BF-TEM and **(D–G)** elemental mapping images of the YMO-SO.

Figure 4A displays the TEM image of the YMO-SO. It can also be seen from the figure that the adhesion and agglomeration between particles is very obvious, and the average particle size is about 40 nm, which is consistent with SEM observation. Figure 4B displays the HRTEM image of the YMO-SO. The interplanar spacing of 0.57, 0.27 and 0.17 nm can be indexed to the (002), (112) and (300) crystal planes of YMO, respectively. Figures 4C–G displays the BF-TEM and elemental mapping images of the YMO-SO. The S element is evenly distributed on the YMO matrix, indicating that the S-O functional group is decorated on the YMO surface.

### 3.3 Optical properties

**FIGURE 5:**
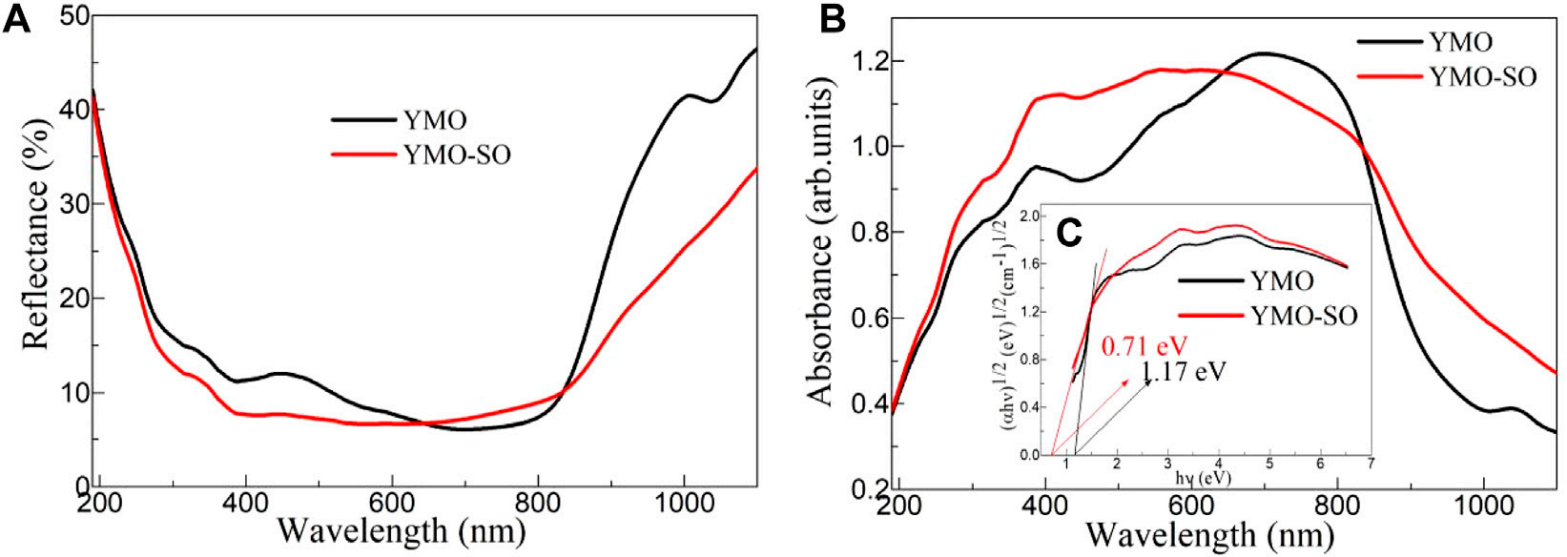
**(A)** UV-vis diffuse reflection spectra, **(B)** UV-vis absorption spectra and **(C)** E.g., values of the YMO and YMO-SO.

The exploration of optical properties of photocatalyst can provide an effective insight into its photoresponse capacity. Figure 5A displays the UV-vis diffuse reflection spectra of the YMO and YMO-SO. At 190–800 nm, the reflectance of all samples decreased with the increasing of the wavelength, but the opposite trend appeared at 800–1,100 nm. According to the literature (Peymannia et al., 2014) and Eqs 2–6, the color parameters (L*, a*, b*, H^o^, ΔE_CIE_^*^) of the YMO and YMO-SO can be evaluated and given in Table 1.

$$\left\{\begin{array}{l} L^{*}=116\times {\left(\frac{Y_{10}}{Y_{n}}\right)}^{\frac{1}{3}}-16,\frac{Y_{10}}{Y_{n}}>0.008856\\ a^{*}=500\times \left[{\left(\frac{X_{10}}{X_{n}}\right)}^{\frac{1}{3}}-{\left(\frac{Y_{10}}{Y_{n}}\right)}^{\frac{1}{3}}\right],\frac{X_{10}}{X_{n}}>0.008856\\ b^{*}=200\times \left[{\left(\frac{Y_{10}}{Y_{n}}\right)}^{\frac{1}{3}}-{\left(\frac{Z_{10}}{Z_{n}}\right)}^{\frac{1}{3}}\right],\frac{Z_{10}}{Z_{n}}>0.008856\\ \left(X_{n},Y_{n},Z_{n}\right)=\left(94.81,100,107.32\right) \end{array}\right.$$ (2)

**TABLE 1:** L*, a*, b*, H^o^, ΔE_CIE_^*^ and, E.g., values of the YMO and YMO-SO.

Sample	Color coordinates						E.g., value (eV)
	L*	a*	b*	c*	H^o^	ΔE_CIE_^*^	
YMO	45.221	−1.958	−4.321	4.744	87.456	45.469	1.17
YMO-SO	37.554	−2.168	−3.421	4.050	57.636	37.772	0.71

The (X_10_, Y_10_, Z_10_) can be described by Eq. 3.

$$\left\{\begin{array}{l} X_{10}=\frac{100\times \sum R\left(\lambda \right)\times S\left(\lambda \right)\times x_{10}\left(\lambda \right)\times \Delta \lambda }{\sum S\left(\lambda \right)\times y_{10}\left(\lambda \right)\times \Delta \lambda }\\ Y_{10}=\frac{100\times \sum R\left(\lambda \right)\times S\left(\lambda \right)\times y_{10}\left(\lambda \right)\times \Delta \lambda }{\sum S\left(\lambda \right)\times y_{10}\left(\lambda \right)\times \Delta \lambda }\\ Z_{10}=\frac{100\times \sum R\left(\lambda \right)\times S\left(\lambda \right)\times z_{10}\left(\lambda \right)\times \Delta \lambda }{\sum S\left(\lambda \right)\times y_{10}\left(\lambda \right)\times \Delta \lambda } \end{array}\right.$$ (3)

Where, λ is the test wavelength, (x_10_(λ), y_10_(λ), z_10_(λ)) is the color matching function, S(λ) is the relative spectral power distribution, R(λ) is the reflectance of the YMO and YMO-SO and △λ = 1 nm is the wavelength interval.

$$H^{o}=\arctan\left(b^{*}/a^{*}\right)$$ (4)

$$\Delta E_{CIE}^{*}=\sqrt{{\left(L^{*}\right)}^{2}+{\left(a^{*}\right)}^{2}+{\left(b^{*}\right)}^{2}}$$ (5)

$$c^{*}=\sqrt{{\left(a^{*}\right)}^{2}+{\left(b^{*}\right)}^{2}\;}$$ (6)

According to the calculation and the results in Table 1, it can be seen that the value of L* is below 50, indicating that the color of the sample tends to be black. The a* and b* values of both samples are less than 0, indicating the presence of certain green and blue components in the samples. The values of L*, a* and c* of YMO-SO are smaller than those of YMO, while the values of b* are larger than YMO. The results show that the color properties of YMO are greatly changed after heat treatment with hydrochloric acid.

According to the diffuse reflection spectrum and Kubelka-Munk (K-M) formula (7) (Yin et al., 2024), the UV-VIS, absorption spectrum of the YMO, and YMO-SO, can be obtained, as shown in Figure 5B.

$$F\left(R\right)=\frac{\alpha }{S}=\frac{{\left(1-R_{\infty }\right)}^{2}}{2R}$$ (7)

Where, R is the reflectance of the YMO and YMO-SO, α is the absorption coefficient of the YMO and YMO-SO, and S is the scattering coefficient of the YMO and YMO-SO. As can be seen from Figure 5B, the UV-VIS absorption spectrum shows the opposite trend to the diffuse reflection spectrum, with the absorption coefficient increasing with the wavelength at 190–800 nm and decreasing with the wavelength at 800–1,100 nm. The absorption coefficients of YMO-SO samples were all larger than those of YMO, except for the range of 680–820 nm. The results confirm that the YMO and YMO-SO samples have high optical absorption coefficients in the 190–1,100 nm range, suggesting that they have UV, visible and near-infrared photocatalytic capabilities.

The relationship between (αhν)^1/2^ and hν can be described by Eq. 8 (Ahmed et al., 2024).

$${\left(F\left(R\right)E\right)}^{n}=A\left(E-E_{g}\right)$$ (8)

where E = hν is the photon energy, A is a proportionality constant, and n = 1/2 for the indirect band gap semiconductor. The relationship between (αhν)^1/2^ and hν for the YMO and YMO-SO samples as shown in Figure 5C. The optical band gap (E.g.,) values of YMO and YMO-SO can be obtained by calculating the intersection of the slope at the steepest part of the curve and the horizontal coordinate. This result is also shown in Table 1. The E.g., values of YMO and YMO-SO are 1.17 and 0.71 eV, respectively. Compared with the YMO sample, the optical band gap value of YMO-SO was significantly decreased.

### 3.4 Photocatalytic activity

#### 3.4.1 Effect of different light sources on the photocatalytic activity

**FIGURE 6:**
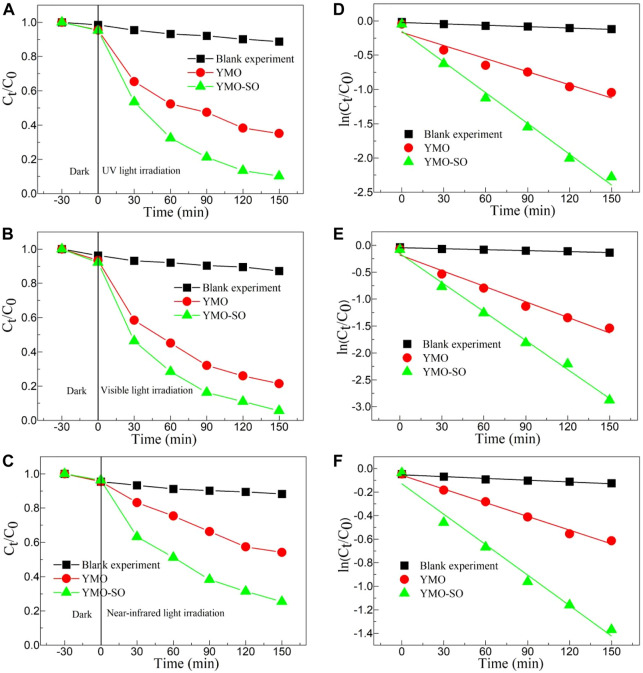
The curves of C_t_/C_0_∼t for the blank experiment, YMO, YMO-SO under **(A)** UV irradiation, **(B)** visible light irradiation and **(C)** near-infrared light irradiation. The curves of ln(C_t_/C_0_) ∼t for the blank experiment, YMO, YMO-SO under **(D)** UV irradiation, **(E)** visible light irradiation and **(F)** near-infrared light irradiation.

UV-Vis absorption spectra of YMO and YMO-SO show that the YMO and YMO-SO samples have high optical absorption coefficients in the ultraviolet, visible and near-infrared ranges, suggesting that they have high photocatalytic activity to degrade pollutants. To explore the photocatalytic activity of YMO and YMO-SO, a photocatalytic experiment was performed with ibuprofen as the target to degrade pollutants. Meanwhile, in order to study the effect of different light on the photocatalytic activity of YMO and YMO-SO photocatalysts, ultraviolet light, visible light and near-infrared light were obtained by adding different filters in front of xenon lamps to study the effect of different light on their photocatalytic activity. The curves of C_t_/C_0_∼t for the blank experiment, YMO, YMO-SO under UV irradiation, visible light irradiation and near-infrared light irradiation as displayed in Figures 6A–C. Performing blank experiments under different lighting conditions found that ibuprofen was almost impossible to degrade. Under the irradiation of three different light sources, YMO-SO showed higher photocatalytic activity than YMO. Obviously, under visible light conditions, YMO-SO demonstrated the best photocatalytic efficiency for the degradation of ibuprofen. Notably, YMO-SO also demonstrated high near-infrared photocatalytic activity for the degradation of ibuprofen.

To more directly reflect the photocatalytic rate of the photocatalyst, the first-order kinetic formula (9) is a very reliable choice (Leelert et al., 2024).

$$1n\left(C_{t}/C_{0}\right)=-\text{kt}$$ (9)

Where, t is the light irradiation time, k is the first-order kinetic rate constant, C_0_ is the initial concentration of ibuprofen, and C_t_ is the concentration of ibuprofen at time t. Figures 6D–F displays the curves of ln(C_t_/C_0_) ∼t for the blank experiment, YMO, YMO-SO under UV irradiation, visible light irradiation and near-infrared light irradiation. Both blank and photocatalytic experiments show a high linear dependence between ln(C_t_/C_0_) and t. Under ultraviolet irradiation, the k values of the blank experiment, YMO and YMO-SO are 6.67922 × 10^−4^, 0.00638 and 0.01496 min^-1^, respectively. The degradation rate of YMO-SO was 2.34 times that of YMO. The k values of the blank experiment, YMO and YMO-SO under visible light irradiation are 6.02804 × 10^−4^, 0.00965 and 0.01794 min^-1^, respectively. The degradation rate of YMO-SO is 1.86 times that of YMO, which seems to be lower than that of UV, but the main reason is that YMO has higher photocatalytic activity under visible light than UV light. When performing the NIR photocatalytic experiment, the k values of the blank experiment, YMO and YMO-SO are 5.04754 × 10^−4^, 0.00388 and 0.00862 min^−1^, respectively. The degradation rate of YMO-SO was 2.22 times that of YMO. It can be seen that the photocatalytic activity of YMO-SO is greatly improved under ultraviolet and NIR light conditions.

#### 3.4.2 Effect of environmental parameters on the photocatalytic activity

**FIGURE 7:**
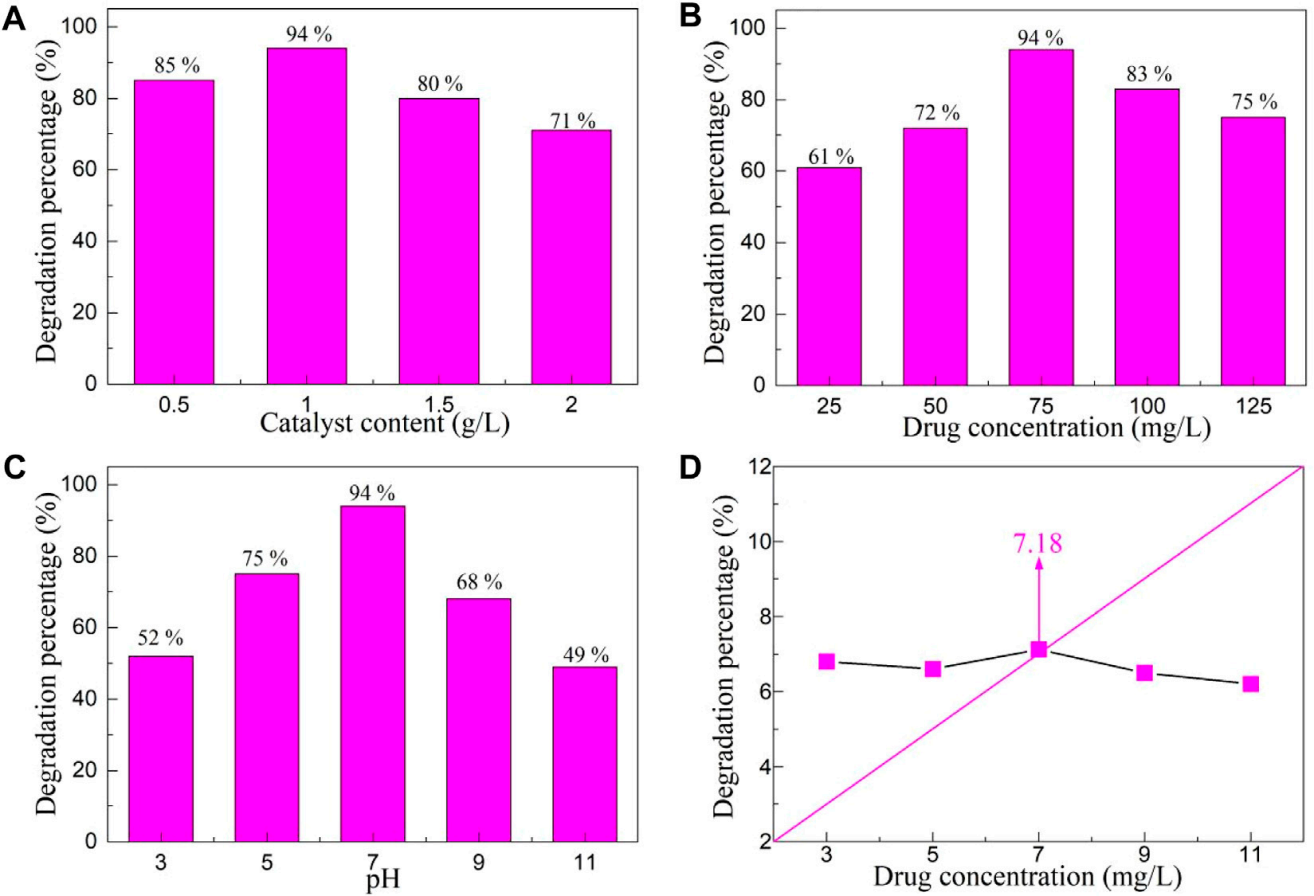
Effects of **(A)** catalyst content, **(B)** drug concentration and **(C)** pH value on the degradation percentage of YMO-SO. **(D)** The point of zero charge (PZC) of YMO-SO.

Various environmental parameters have a great impact on the photocatalytic activity of the photocatalyst, so it is particularly important to explore the effect of environmental parameters on the photocatalytic activity of YMO-SO photocatalyst. Since the degradation percentage of YMO-SO was the highest under visible light irradiation, the photocatalytic activity of YMO-SO was studied with visible light as the research object. Figure 7A displays the effect of catalyst content on the degradation percentage of YMO-SO. The range of catalyst content is selected from 0.5–2 g/L, and other conditions are unchanged during the execution of this experiment. The illumination time was 150 min, the drug concentration was 75 mg/L and pH = 7. With the increase of catalyst content, the degradation percentage of YMO-SO first increased and then decreased. The photocatalytic activity of YMO-SO is low due to the ineffective utilization of the active site in the catalyst when the content is too low. However, when the catalytic content is too high, the photocatalytic activity of YMO-SO photocatalyst decreases, mainly because of the light penetration of suspension decreased (Kumari et al., 2021; Han et al., 2024). The results showed that the optimum catalyst content for the degradation of ibuprofen with YMO-SO photocatalyst was 1 g/L.

Figure 7B displays the effect of drug concentration on the degradation percentage of YMO-SO. Similarly, other parameters remain unchanged, only the drug concentration range is changed to 25–125 mg/L. With the increase of drug concentration, the photocatalytic degradation percentage of YMO-SO photocatalyst first increased and then decreased. Similar to the catalyst content, when the drug concentration is low, the surface active site in the YMO-SO photocatalyst is not effectively utilized, so the percentage of photocatalytic degradation is not high. When the drug concentration reaches 100 mg/L, the degradation percentage is significantly lower than that at 75 mg/L, mainly because the high drug concentration increases the path length of the photon entering a solution containing the catalyst and the drug (Otálvaro-Marín et al., 2017; Wang et al., 2024). In subsequent photocatalytic experiments, the optimal drug concentration of the YMO-SO photocatalyst for the degradation of ibuprofen was determined to be 75 mg/L.

Figure 7C displays the effect of pH value on the degradation percentage of YMO-SO. Under different pH conditions, the surface of the catalyst exhibits different types of charges, and the drug also exhibits different electrical properties, which will make the pH value have a great impact on the catalytic activity of the catalyst. In Figure 7C, with the increase of pH value, the photocatalytic activity of YMO-SO first increased and then decreased. It is necessary to test the point of zero charge (PZC) of the photocatalyst in order to deeply understand the effect of pH on the photocatalytic activity of the photocatalyst. Figure 7D displays the point of PZC value of YMO-SO. By measuring the pH value of the reaction solution before and after photocatalysis, the PZC value of the YMO-SO photocatalyst was 7.18. When the amount of drug adsorbed on the surface of the YMO-SO, on the basis of Eqs 10, 11, the pH value lower than the PZC of the YMO-SO, the surface of the YMO-SO becomes positive charge and it is the negative charge for pH *>* PZC.

$$\text{pH }<\text{ PZC}:\left(\text{YMO}-\text{SO}\right)-\text{OH}+H^{+}\;\Leftrightarrow \;\left(\text{YMO}-\text{SO}\right){\text{OH}}_{2}^{+}$$ (10)

$$\text{pH }>\text{PZC}:\left(\text{YMO}-\text{SO}\right)-\text{OH}+{\text{OH}}^{-}\;\Leftrightarrow \text{YMO}-{\text{SO}}^{-}+H_{2}O$$ (11)

At a pH of 7, YMO-SO showed the best degradation percentage. It can be seen that the ibuprofen is preferentially degraded by YMO-SO under neutral conditions.

#### 3.4.3 Capture experiments, ESR/EPR experiments and Mott- Schottky curves

**FIGURE 8:**
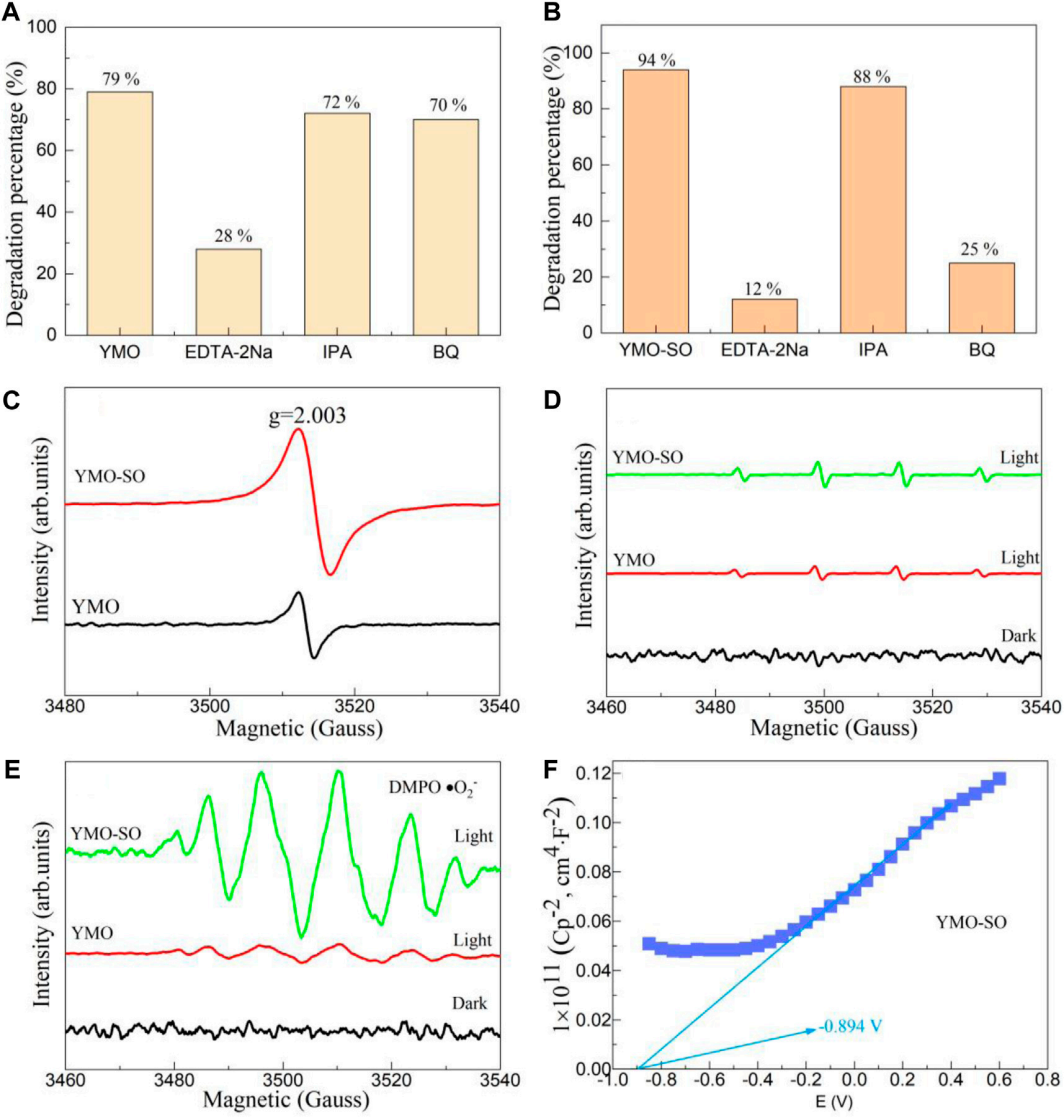
Capture experiments of the **(A)** YMO and **(B)** YMO-SO. **(C)** EPR spectrum for the h_VB_^+^ and ESR spectra for the **(D)** •OH and **(E)** •O_2_^−^ of the YMO and YMO-SO. **(F)** The Mott- Schottky (M–S) curves of YMO-SO.

The main functions of photocatalysts in the degradation of pollutants are the h_VB_^+^ and active free radicals. In order to explore the role of h_VB_^+^ and active radicals in the photocatalysis process, capture experiments must be performed. Figures 8A, B displays the capture experiments of the YMO and YMO-SO for the degradation of ibuprofen under visible light irradiation, respectively. When the capture experiment of YMO was performed (Figure 8A), the degradation percentage of YMO photocatalyst after adding EDTA-2Na, IPA and BQ was 28%, 72% and 70%, respectively. The results showed that only h_VB_^+^ played a major role in the photocatalytic process, while •OH and •O_2_^−^ hardly participated in the degradation of ibuprofen. The capture experiments of the YMO-SO as shown in Figure 8B. When EDTA-2Na, IPA and BQ were added to the reaction solution, the degradation percentages were 12%, 88% and 25%, respectively. The results showed that the •OH and •O_2_^−^ played major role in the degradation of ibuprofen by YMO-SO.

To further confirm the dominance of the h_VB_^+^, •OH and •O_2_^−^ in the degradation of ibuprofen by YMO-SO photocatalyst, ESR/EPR experiments were performed and shown in Figures 8C–E. For the YMO and YMO-SO samples, a obvious peak at g = 2.003 can be observed in Figure 8C. These results confirmed that the h_VB_^+^ played an important role in YMO and YMO-SO photocatalysts for the degradation of ibuprofen. In Figure 8D, although both YMO and YMO-SO samples showed spikes under light irradiation conditions, the •OH played little role in the photocatalysis process. When the •O_2_^−^ validation experiment was performed, it was found that there were six obvious characteristic peaks of YMO-SO, indicating that the •O_2_^−^ played a major role. The capture experiments and ESR/EPR experiments confirmed that the h_VB_^+^ played a major role in the degradation of ibuprofen by YMO, while the h_VB_^+^ and •O_2_^−^ played a major role in the degradation of ibuprofen by YMO-SO.

Generally, YMO is a p-type semiconductor (Balamurugan and Lee, 2015; Turut et al., 2019). When the YMO is treated with sulfuric acid, it may be converted into an n-type semiconductor. Therefore, the Mott-Schottky curve can be used to confirm this hypothesis. Figure 8F displays the Mott- Schottky (M-S) curves of YMO-SO. The slope of the M-S curve is positive, indicating that YMO-SO is an n-type semiconductor, and the results are consistent with the hypothesis. According to the experimental results, the flat band potential (V_FB_) of YMO-SO photocatalyst is −0.894 V. For the n-type semiconductor, the conduction band potential at normal hydrogen electrode (NHE) can be described by Eq. 12.

$$V\left(\text{NHE}\right)=V_{\text{FB}}+0.059\text{pH}+0.242$$ (12)

According to the calculation, the conduction potential of YMO-SO is −0.18 V. This result will help to analyze the photocatalytic mechanism of YMO-SO.

#### 3.4.4 Cyclic stability experiments

**FIGURE 9:**
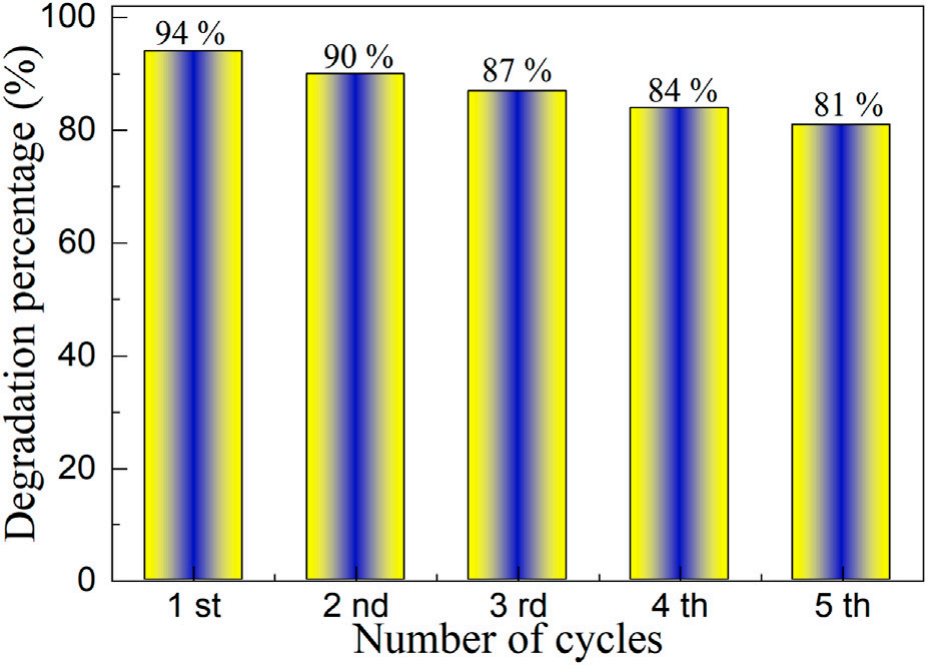
Cyclic stability experiment of YMO-SO photocatalyst.

The goal of the final industrial application of photocatalyst is to be used repeatedly. To study the cyclic stability of YMO-SO photocatalyst, Figure 9 describes the cyclic stability experiment of YMO-SO photocatalyst. Before each cycle experiment, it is necessary to centrifuge, filter, dry and sintering the catalyst used last time before performing the next cycle stability experiment. After five cycles of stability experiments, the degradation percentage of YMO-SO photocatalyst decreased from 94% to 81%. The loss of both the photocatalyst during use and the active site during photocatalysis means this reduction is acceptable and confirms the recyclability of the YMO-SO photocatalyst.

### 3.5 Photocatalytic mechanism

According to the results of capture experiment and free radical detection experiment, the h_VB_^+^ played a major role for YMO and YMO-SO in the entire photocatalytic process, and the •O_2_^−^ also played a major role in the degradation of ibuprofen by YMO-SO. However, why •OH and •O_2_^−^ play different roles in different samples during the experiment needs to be explored in detail in combination with the band theory. According to the band theory, the conduction band potential (*E*_CB_) and valence band potential (*E*_VB_) of YMO are calculated on the basis of Eqs 13, 14 (You et al., 2020; Dang et al., 2024; Zhu et al., 2024).

$$E_{\text{CB}}=X\;-\;E^{e}\;-\;0.5E_{g}$$ (13)

$$E_{\text{VB}}=E_{\text{CB}}+E_{g}$$ (14)

Where, *E* ^e^ = 4.5 eV. The X = 5.52 of the YMO can be calculated by Eq. 15.

$$X\left(YMnO_{3}\right)=\sqrt[{5}]{X\left(Y\right)X\left(Mn\right)X{\left(O\right)}^{3}}$$ (15)

**FIGURE 10:**
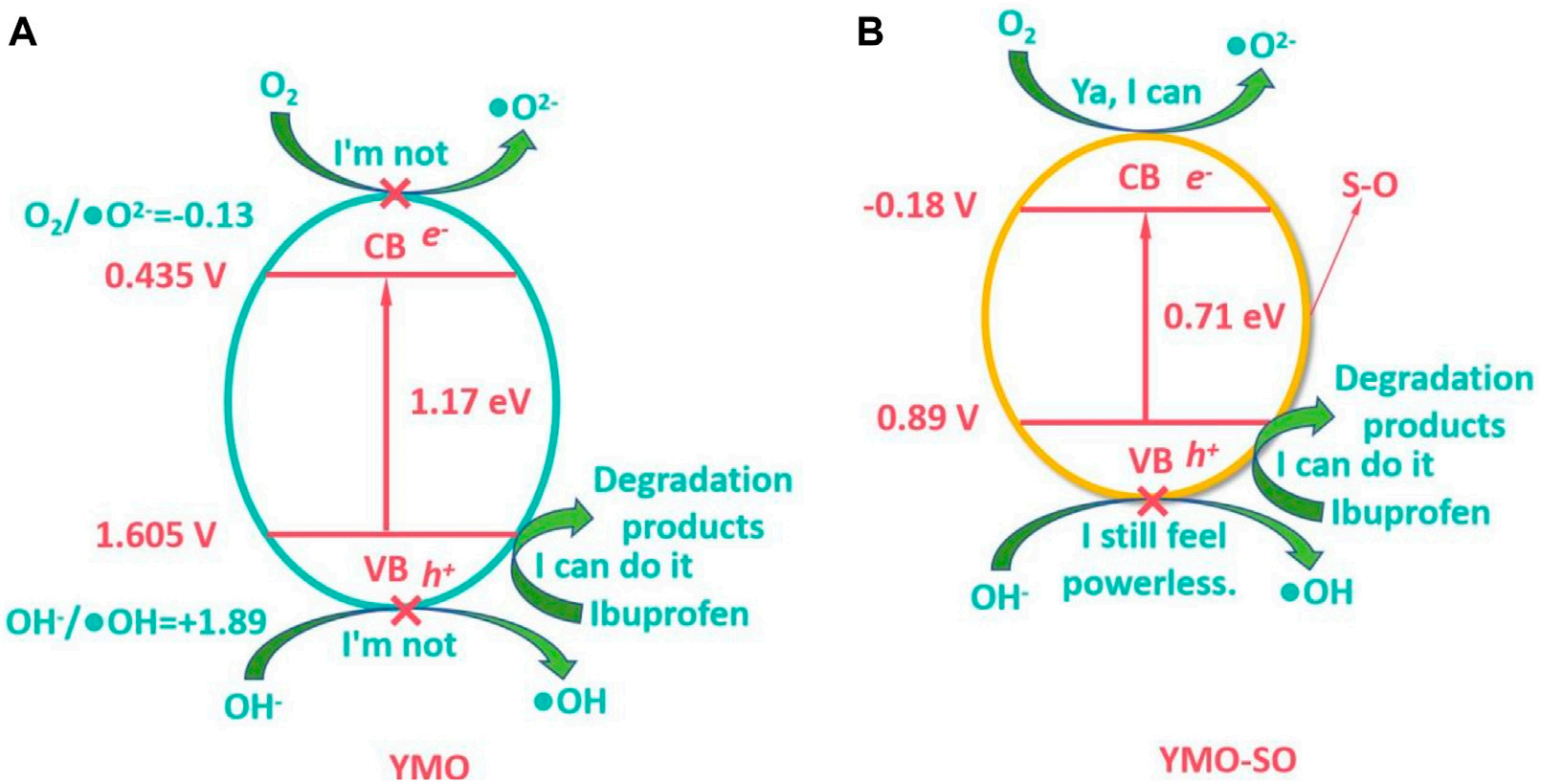
Photocatalytic mechanism of the **(A)** YMO and **(B)** YMO-SO.

Where, X(Y) = 3.19 eV, X(Mn) = 3.75 eV and X(O) = 7.54 eV. YMO and NO have an The *E*_CB_ and *E*_VB_ of YMO are 0.435 and 1.605 V, respectively. According to calculation, Figure 10A displays the photocatalytic mechanism of the YMO. The redox potentials of O_2_/•O_2_^−^ and OH^−^/•OH were −0.13 and +1.89 V, respectively, while the E_CB_ and E_VB_ of YMO could not reach the lower limit of obtaining •OH and •O_2_^−^ so the •OH and •O_2_^−^ in YMO were almost not involved in the reaction. Thus, only the h_VB_^+^ is involved in the photocatalytic reaction to degrade ibuprofen into non-toxic products.

Since the content of S-O bond in YMO-SO cannot be accurately determined, it is not practical to calculate its conduction band and valence band potential. It can be obtained from Figure 8F that the conduction band potential of YMO-SO is −0.18 V, and then its valence band potential is 0.89 V. According to the calculated results, the photocatalytic mechanism of YMO-SO as shown in Figure 10B. The results show that the •O_2_^−^ can be produced in YMO-SO, and the •O_2_^−^ will directly react with ibuprofen to produce non-toxic products. Simultaneously, the h_VB_^+^ will also interact with ibuprofen to produce non-toxic products.

## 4 Conclusion

The YMO and YMO-SO photocatalysts were synthesized by the oxalate complexation and sulfuric acid heat treatment method. The crystal structure of YMO was not changed after heat treatment with sulfuric acid, but the particle size of YMO was slightly increased by a layer of S-O bond on its surface modification. Simultaneously, heat treatment of YMO with sulfuric acid changes its color properties and decreases its optical band gap value. The photocatalytic activity of the YMO-SO photocatalyst was studied under different illumination conditions and different environmental parameters. The photocatalytic activity of the YMO-SO photocatalyst was studied under ultraviolet, visible and near-infrared light. The optimum catalyst content of YMO-SO photocatalyst for the degradation of ibuprofen is 1 g/L, drug concentration is 75 mg/L and pH is 7. According to the capture experiment, free radical detection experiment and energy band theory, it was determined that the h_VB_^+^ and •O_2_^−^ played the main roles in the degradation of ibuprofen by YMO-SO photocatalyst. This novel work will provide technical support for synthesizing other novel photocatalysts and studying their photocatalytic activities.

## Data Availability

The raw data supporting the conclusion of this article will be made available by the authors, without undue reservation.

## References

[B1] Abdo S. M. El-Hout S. I. Rashed M. N. El-Dosoqy T. I. El-Sheikh S. M. (2024). Boosting visible-light photodegradation of methyl orange and ibuprofen over rGO-supported Ag_3_PO_4_ nanocomposite. Inorg. Chem. Commun. 161, 112035. 10.1016/j.inoche.2024.112035

[B2] Ahmed M. Abdel-Aty A. H. Alshehri K. Alshahrie A. Al-Harbi N. Shaaban E. R. (2024). Structure, optical properties, and electrical properties of low copper-doped cadmium oxide thin films for optoelectronic applications. Indian J. Phys., 1–10. 10.1007/s12648-024-03167-7

[B3] Balamurugan C. Lee D. W. (2015). Perovskite hexagonal YMnO_3_ nanopowder as p-type semiconductor gas sensor for H_2_S detection. Sensors Actuators B Chem. 221, 857–866. 10.1016/j.snb.2015.07.018

[B4] Cao Z. Wang C. Chen J. (2018). Synthesis and photocatalytic property of p–n junction YMnO_3_/SrTiO_3_ composites. Mater. Res. Express 5 (11), 115512. 10.1088/2053-1591/aadec0

[B5] Dang M. Guo Y. Tian Y. (2024). Preparation and study of p–n junction YMnO_3_/TiO_2_ composite photocatalysts for the degradation of tetracycline hydrochloride. J. Mater. Sci. Mater. Electron. 35 (3), 221. 10.1007/s10854-024-11980-3

[B6] Falletta E. Galloni M. G. Mila N. bin Roslan M. N. Abd Ghani N. Cerrato G. (2023). Fast and efficient piezo-photocatalytic mineralization of ibuprofen by BiOBr nanosheets under solar light irradiation. ACS Photonics 10 (11), 3929–3943. 10.1021/acsphotonics.3c00724

[B7] Han C. Kundu B. K. Liang Y. Sun Y. (2024). Near-infrared light-driven photocatalysis with an emphasis on two-photon excitation: concepts, materials, and applications. Adv. Mater. 36 (5), 2307759. 10.1002/adma.202307759 37703435

[B8] Jin H. Xu X. Liu R. Wu X. Chen X. Chen D. (2024). Electro-oxidation of Ibuprofen using carbon-supported SnO_x_-CeO_x_ flow-anodes: the key role of high-valent metal. Water Res. 252, 121229. 10.1016/j.watres.2024.121229 38324989

[B9] Kumar R. D. Thangappan R. Jayavel R. (2019). Structural, morphological and photocatalytic activity of YMnO_3_ nanorods. J. Nanosci. Nanotechnol. 19 (4), 2385–2390. 10.1166/jnn.2019.15806 30487005

[B10] Kumari P. Bahadur N. O'Dell L. A. Kong L. Sadek A. Merenda A. (2021). Nanoscale 2D semi-conductors–Impact of structural properties on light propagation depth and photocatalytic performance. Sep. Purif. Technol. 258, 118011. 10.1016/j.seppur.2020.118011

[B11] Leelert Y. Rojviroon T. Sirivithayapakorn S. Rajendran R. Muangmora R. Akechatree N. (2024). Synergistic effects of micronanobubbles and AC/Ag–TiO_2_ nanocomposites in photocatalytic process. Biocatal. Agric. Biotechnol. 57, 103096. 10.1016/j.bcab.2024.103096

[B12] Li M. Wang S. Gao H. Yin Z. Chen C. Yang H. (2023). Selective removal of antibiotics over MgAl_2_O_4_/C_3_N_4_/YMnO_3_ photocatalysts: performance prediction and mechanism insight. J. Am. Ceram. Soc. 106 (4), 2420–2442. 10.1111/jace.18946

[B13] Li Y. Jin Y. Liu M. Lin Z. Chen Z. Zhuang X. (2024). Rational design of ionic covalent organic frameworks for PPCPs elimination: insights into ibuprofen adsorption performance and mechanism. Colloids Surfaces A Physicochem. Eng. Aspects 685, 133309. 10.1016/j.colsurfa.2024.133309

[B14] López-Alvarez M. Á. Silva-Jara J. M. Silva-Galindo J. G. Reyes-Becerril M. Velázquez-Carriles C. A. Macías-Rodríguez M. E. (2023). Determining the photoelectrical behavior and photocatalytic activity of an h-YMnO_3_ new type of obelisk-like perovskite in the degradation of malachite green dye. Molecules 28 (9), 3932. 10.3390/molecules28093932 37175343 PMC10179874

[B15] Munisha B. Mishra B. Nanda J. Sahoo N. K. Ghosh D. Sankaran K. J. (2023). Enhanced photocatalytic degradation of 4-nitrophenol using polyacrylamide assisted Ce-doped YMnO_3_ nanoparticles. J. Rare Earths 41 (10), 1541–1550. 10.1016/j.jre.2022.11.022

[B16] Navrozidou E. Melidis P. Ntougias S. (2019). Biodegradation aspects of ibuprofen and identification of ibuprofen-degrading microbiota in an immobilized cell bioreactor. Environ. Sci. Pollut. Res. 26, 14238–14249. 10.1007/s11356-019-04771-5 30859445

[B17] Osman A. I. Ayati A. Farghali M. Krivoshapkin P. Tanhaei B. Karimi-Maleh H. (2024). Advanced adsorbents for ibuprofen removal from aquatic environments: a review. Environ. Chem. Lett. 22 (1), 373–418. 10.1007/s10311-023-01647-6

[B18] Otálvaro-Marín H. L. Mueses M. A. Crittenden J. C. Machuca-Martinez F. (2017). Solar photoreactor design by the photon path length and optimization of the radiant field in a TiO_2_-based CPC reactor. Chem. Eng. J. 315, 283–295. 10.1016/j.cej.2017.01.019

[B19] Othman S. I. Shemy M. H. Alfassam H. E. Alqhtani H. A. Allam A. A. Abukhadra M. R. (2024). Insight into the catalytic performance of a zinc-pillared curcumin/bentonite composite for enhanced oxidation of ibuprofen residuals into environmental products: the pathway and toxicity. Catalysts 14 (2), 129. 10.3390/catal14020129

[B20] Peymannia M. Soleimani-Gorgani A. Ghahari M. Najafi F. (2014). Production of a stable and homogeneous colloid dispersion of nano CoAl_2_O_4_ pigment for ceramic ink-jet ink. J. Eur. Ceram. Soc. 34 (12), 3119–3126. 10.1016/j.jeurceramsoc.2014.03.022

[B21] Ramesh S. Yuen T. F. Shen C. J. (2008). Conductivity and FTIR studies on PEO–LiX [X: CF_3_SO_3_ ^−^, SO_4_ ^2−^] polymer electrolytes. Spectrochimica Acta Part A Mol. Biomol. Spectrosc. 69 (2), 670–675. 10.1016/j.saa.2007.05.029 17600757

[B22] Rebollo B. Jiménez A. Trujillano R. Rives V. Gil A. Vicente M. A. (2024). Hydrocalumite–TiO_2_ hybrid systems synthesized from aluminum salt cake for photodegradation of ibuprofen. J. Environ. Chem. Eng. 12 (2), 112395. 10.1016/j.jece.2024.112395

[B23] Shakir I. (2024). Synthesis of Gd doped BiFeO_3_/g-C_3_N_4_ composite: enhancement of solar mediated photocatalytic performance. Mater. Sci. Eng. B 302, 117252. 10.1016/j.mseb.2024.117252

[B24] Shukla J. Saxena P. Joshi P. Joshi P. Mishra A. (2023). Impact of aliovalent ions doping on structural and electrical characteristics of YMnO_3_ ceramic. Appl. Phys. A 129 (10), 731. 10.1007/s00339-023-07009-x

[B25] Sruthi L. Janani B. Khan S. S. (2021). Ibuprofen removal from aqueous solution via light-harvesting photocatalysis by nano-heterojunctions: a review. Sep. Purif. Technol. 279, 119709. 10.1016/j.seppur.2021.119709

[B26] Tong H. J. Reid J. P. Dong J. L. Zhang Y. H. (2010). Observation of the crystallization and supersaturation of mixed component NaNO_3_− Na_2_SO_4_ droplets by FTIR-ATR and Raman spectroscopy. J. Phys. Chem. A 114 (46), 12237–12243. 10.1021/jp1080548 21028770

[B27] Turut A. Coșkun M. Coșkun F. M. Polat O. Durmuș Z. Çağlar M. (2019). The current-voltage characteristics of the ferroelectric p-YMnO_3_ thin film/bulk p-Si heterojunction over a broad measurement temperature range. J. Alloys Compd. 782, 566–575. 10.1016/j.jallcom.2018.12.246

[B28] Wang H. Cheng Y. Zhu J. Zhang L. (2024). Photon management enabled by opal and inverse opal photonic crystals: from photocatalysis to photoluminescence regulation. ChemPlusChem, e202400002. 10.1002/cplu.202400002 38527947

[B29] Wang S. Li M. Gao H. Yin Z. Chen C. Yang H. (2023). Construction of CeO_2_/YMnO_3_ and CeO_2_/MgAl_2_O_4_/YMnO_3_ photocatalysts and adsorption of dyes and photocatalytic oxidation of antibiotics: performance prediction, degradation pathway and mechanism insight. Appl. Surf. Sci. 608, 154977. 10.1016/j.apsusc.2022.154977

[B30] Wang S. Li M. Yin Z. Gao H. Liu H. Yang H. (2022). Skillfully grafted C-O functional group to enhance the adsorption/photocatalytic mechanism of YMnO_3_/MgAl_2_O_4_ heterojunction photocatalysts. Adv. Powder Technol. 33 (11), 103771. 10.1016/j.apt.2022.103771

[B31] Wang S. F. Yang H. Xian T. Liu X. Q. (2011). Size-controlled synthesis and photocatalytic properties of YMnO_3_ nanoparticles. Catal. Commun. 12 (7), 625–628. 10.1016/j.catcom.2010.11.023

[B32] Wang Y. Song J. (2020). Synthesized and photocatalytic mechanism of the NiO supported YMnO_3_ nanoparticles for photocatalytic degradation of the methyl orange dye. Z. Für Phys. Chem. 234 (1), 153–170. 10.1515/zpch-2019-1392

[B33] Wang Y. Tian H. (2020). Study on the construction of YMnO_3_/CeO_2_ composite photocatalyst heterostructure and photocatalytic degradation of methyl red. Optik 201, 163524. 10.1016/j.ijleo.2019.163524

[B34] Wu Y. Zhou X. Li M. Wang Y. Zhou B. Wu N. (2019). 2D/3D interface engineering: direct Z-scheme g-C_3_N_4_/YMnO_3_ heterojunction for reinforced visible-light photocatalytic oxidation. J. Mater. Sci. Mater. Electron. 30, 17601–17611. 10.1007/s10854-019-02109-y

[B35] Yin Y. Zhang Y. Huang Z. Xin H. Hu X. Gao Y. (2024). A kinetic research method for coal oxidation based on the Kubelka-Munk equation and the Starink method. Combust. Sci. Technol. 196 (2), 245–260. 10.1080/00102202.2022.2072685

[B36] You J. Zhan S. Wen J. Ma Y. Zhu Z. (2020). Construction of heterojunction of Ag_2_S modified yttrium manganate visible photocatalyst and study on photocatalytic mechanism. Optik 217, 164900. 10.1016/j.ijleo.2020.164900

[B37] Yulizar Y. Abdullah I. Surya R. M. Alifa N. L. (2023). Green synthesis of novel YMnO_3_-doped TiO_2_ for enhanced visible-light-driven photocatalytic degradation of malachite green. J. Environ. Manag. 342, 118139. 10.1016/j.jenvman.2023.118139 37285771

[B38] Zhang H. Lü X. M. Ding J. L. Xie J. M. Yan C. H. (2011). Synthesis and performance of perovskite YMn_x_Fe_1-x_O_3_ photocatalyst. Mater. Sci. Forum 675, 1025–1029. 10.4028/scientific.net/MSF.675-677.1025

[B39] Zhang X. Liu X. Wang Y. Tong B. Zhang J. (2022). Study on photocatalytic activity of cage-like PAM/YMnO_3_ composite photocatalyst. Russ. J. Phys. Chem. A 96 (14), 3103–3110. 10.1134/S0036024423020310

[B40] Zhang Z. Wang S. (2017). High-temperature phase transition, coordination mechanism and magnetism in multiferroic YMnO_3_ nanopowders. J. Mater. Sci. Mater. Electron. 28, 10940–10950. 10.1007/s10854-017-6874-x

[B41] Zhu J. Cheng X. Cui Y. Chen F. (2024). Photocatalytic activity and mechanism of YMnO_3_/NiO photocatalyst for the degradation of oil and gas field wastewater. Front. Chem. 12, 1408961. 10.3389/fchem.2024.1408961 38752200 PMC11094212

